# Variations in sagittal locations of anterior cruciate ligament tibial footprints and their association with radiographic landmarks: a human cadaveric study

**DOI:** 10.1186/s12891-017-1822-8

**Published:** 2017-11-14

**Authors:** Hyung Joon Cho, Tae Kyun Kim, Seung-Baik Kang, Min Uk Do, Chong Bum Chang

**Affiliations:** 1 Department of Orthopaedic Surgery, Pusan National University Yangsan Hospital, 20 Geumo-ro, Mulgeum-eup, Gyeongsangnam-do, Yangsan-si, 50612 South Korea; 2 Joint Reconstruction Center, Seoul National University Bundang Hospital, 82, Gumi-ro 173beon-gil, Bundang-gu, Seongnam-si, Gyeonggi-do 13620 South Korea; 3 Department of Orthopaedic Surgery, Seoul National University Boramae Hospital, 20, Boramae-ro 5-gil, Dongjak-gu, Seoul, 07061 South Korea

**Keywords:** ACL tibial attachment, Full extension lateral radiograph, Blumensaat’s line

## Abstract

**Background:**

This cadaveric study aimed to demonstrate variation of the anterior cruciate ligament (ACL) tibial attachment in the sagittal plane, and to analyze the radiographic landmarks which predict the sagittal location of the ACL tibial attachment.

**Methods:**

In 20 cadaveric knees, native ACLs were removed and the centers of the ACL tibial and femoral attachments were marked with metal pins. Full extension lateral radiographs were then obtained in each cadaveric knee. Using the full extension lateral radiographs, the sagittal location of the ACL tibial footprint center was estimated as a percentage in the Amis and Jakob’s line. Several radiographic landmarks including the geometry of Blumensaat’s line and the apex of the tibial eminence were measured. Then, the relationship between the variation of the sagittal location of the ACL tibial footprint and several radiographic landmarks were analyzed using Pearson’s correlation analysis.

**Results:**

The average sagittal position of the native ACL tibial footprint was 40.9% (range: 38.0–45.0%). The line connecting the centers of the ACL footprint was nearly parallel to Blumensaat’s line, with an average angle of 1.7° (range: 0–4.1°). In addition, the distance from the point where Blumensaat’s line meets the tibial articular surface to the center of the ACL tibial footprint was almost consistent, at 7.6 mm on average (range: 6.4–8.7 mm). The correlation analysis revealed that the geometry of Blumensaat’s line was significantly correlated with the sagittal location of the ACL tibial footprint.

**Conclusion:**

The radiographic landmark that showed a significant correlation with the ACL tibial footprint in the full extension lateral radiographs was Blumensaat’s line.

## Background

Appropriate placement of the tibial and femoral tunnels is the most important surgical factor for successful anterior cruciate ligament (ACL) reconstruction [1–6]. However, the ideal tunnel position during ACL reconstruction remains an ongoing debate. Recently, there has been an emphasis on anatomical graft placements to re-create normal physiologic graft tension. Evidence suggests that the kinematics of reconstructed knees can be improved if the tibial and femoral tunnels are centralized within their respective footprints [7, 8]. Therefore, precise knowledge of the normal anatomy of the ACL is crucial for the success of reconstructive surgery, allowing tunnel placement in the proper anatomical locations.

Correct placement of the tibial tunnel is vital for successful surgery and to avoid complications such as anterior knee pain, loss of knee extension, instability and graft impingement [9–12]. In the last decade, concerns regarding possible graft impingement may have led to a more posterior placement of the tibial tunnel during ACL reconstruction, which tends to result in a more vertical graft in the sagittal plane [13–15]. Recently, some clinical and biomechanical studies have suggested that this vertical graft orientation may not control postoperative rotational stability and may be associated with poor subjective and objective outcome scores after ACL reconstruction [12, 16, 17]. Therefore, contemporary ACL reconstruction procedures in the tibial tunnel formation have focused on how to centralize the tunnel within its respective footprint.

In order to accurately place the tibial tunnel during ACL reconstruction, detailed knowledge of the tibial insertion site is imperative. One commonly used method for tibial tunnel placement relies on arthroscopic visualization of the tibial insertion. In acute ACL tears, an arthroscopic surgeon is able to view the tibial remnants of the ACL. However, it is difficult to determine the exact location for the placement of a graft with a relatively smaller diameter within the insertion site. Furthermore, in the setting of a chronic ACL tear or a revision ACL reconstruction, it can be difficult, even for an experienced surgeon, to identify the correct location of the tibial tunnel. In such cases, it may be helpful to use fluoroscopy to verify the anatomic point before drilling of the bone tunnel. Recently, a series of radiographic studies have evaluated methods to determine the sagittal location of the ACL tibial insertion [18–27]. However, the data showed a wide variation according to the anatomical features of the study participants. Thus, these quantitative evaluations failed to yield consistent data or reliable clinical applications [28]. Therefore, a comprehensive, clinically reproducible set of guidelines to assess the radiographic sagittal locations of patient specific ACL tibial insertion is still required.

The purpose of this study was therefore to analyze the radiographic landmarks which predict the sagittal location of the ACL tibial attachment in the cadaveric knee. It was hypothesized that reliable positions for ACL tibial attachments could be established with the use of radiographic landmarks. This information could be used to assist with tunnel placement during arthroscopic anatomic ACL reconstruction and to radiologically confirm correct tunnel placement.

## Methods

### Specimens

Twenty fresh-frozen non-paired cadaveric knees which were donated to our university anatomy program were used in the study (18 men and 2 women; mean age, 57 years; range, 51–68 years; 11 right and 9 left specimens). No specimens had evidence of previous surgery or significant degenerative arthritis. In each specimen, the femur, tibia, and fibula were transected, leaving a minimum length of 20 cm for each bone. Specimens were thawed overnight at room temperature. Before testing, two radiographs were taken (full extension anteroposterior and lateral) in each cadaveric knee. Based on these radiographs, the anteroposterior and mediolateral width of the femur and tibia were measured and used as an index of size for each cadaver. All dissections and markings were performed by a single surgeon (CBC).

### Dissection of specimens

Knee joints were opened using medial parapatellar arthrotomy. After careful dissection of soft tissues, the identified ACL was resected from its femoral and tibial insertions, leaving a remnant “stump” approximately 1 to 2 mm in length. The footprints were outlined, and the center was marked using a permanent ink pen. A guide pin was inserted from the anterior margin of the medial collateral ligament toward the center of the tibial footprint using a 50° ACL tibial guide (Acufex, Smith & Nephew, Memphis, Tenn), until the tip of the guide pin was seen at the center of the tibial footprint. A guide pin was also placed in the center of the femoral footprint using a femoral offset guide (Acufex, Smith & Nephew, Memphis, Tenn). The guide pin was then inserted past the center of the femoral footprint and advanced until the tip of the pin reached the femoral footprint.

### Radiographic assessment

Full extension lateral radiographs were taken in each cadaveric knee. True lateral radiographs were obtained, ensuring that the posterior aspects of the medial and lateral femoral condyles overlapped. A 1 cm × 1 cm radio-opaque grid was included on all radiographs to correct for magnification disparities due to the potential variability in distances between the specimens and the X-ray source. All radiographic images were digitally acquired using a picture archiving and communication system (PACS; Impax: Agfa, Antwerp, Belgium), and assessments were subsequently carried out using the PACS software.

**Fig. 1:**
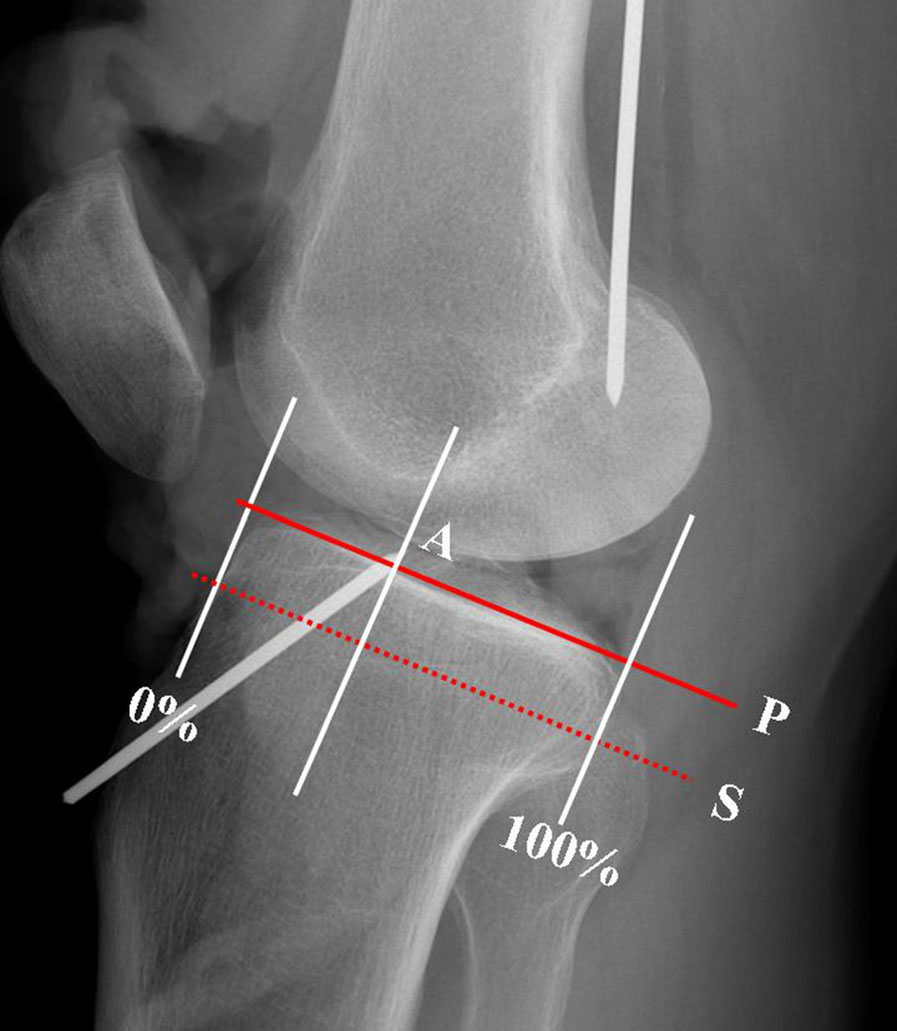
Center of the ACL tibial footprint (A) in Amis & Jakob’s line (line S, dotted line), which is parallel to the medial tibial plateau (line P, solid line) and passing through the posterior corner of the shelf

**Fig. 2:**
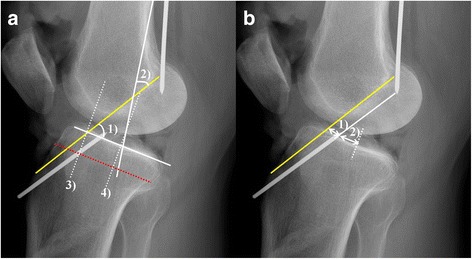
Measurement of radiographic landmarks in full extension lateral radiographs. **a** 1) Angle between Blumensaat’s line and the tibial articular surface, 2) Angle between Blumensaat’s line and the posterior femoral cortex, 3) Sagittal location of Blumensaat’s line in Amis & Jakob’s line, 4) Sagittal location of the apex of the tibial eminence in Amis & Jakob’s line. **b** 1) Distance from Blumensaat’s line to the center of the ACL tibial footprint, 2) Distance from the apex of the tibial eminence to the center of the ACL tibial footprint

Blumensaat’s line and the tibial eminence were identified on the full extension lateral radiograph. The sagittal location of the ACL tibial footprint center was determined using the reference line described by Amis and Jakob (Fig. 1, line S) [29]. This reference line has been used in many radiographic studies regarding the location of ACL since being introduced at a scientific workshop meeting concerning reconstruction of the cruciate ligaments for describing anteroposterior graft position on the tibia [18, 19, 23, 27]. We also used this reference line for better comparison with previous studies. In accordance with this method, a line representing the maximum anteroposterior length of the tibia was drawn parallel to the medial tibial plateau (Fig.1, line P), from the most posterior corner of the proximal tibial plateau to the most anterior tibial margin. The centers of the ACL tibial footprints were orthogonally projected onto the line, and percentage lengths were calculated in the line to 100% from the anterior to the posterior cortex (Fig. 1). We then selected radiographic landmarks that could be easily identified in the full extension lateral radiographs as follows (Fig. 2a, b):

1. Angle between Blumensaat’s line and tibial articular surface.
2. Angle between Blumensaat’s line and posterior femoral cortex.
3. Sagittal location of Blumensaat’s line in Amis and Jakob’s line.
4. Sagittal location of the apex of the tibial eminence in Amis and Jakob’s line.
5. Distance from the center of the ACL tibial footprint to the point where Blumensaat’s line meets the tibial articular surface.
6. Distance from the center of the ACL tibial footprint to the apex of the tibial eminence.
7. Angle between the slope of Blumensaat’s line and the course of the ACL, represented by the line connecting the tips of the femoral and tibial guide pins.

Among these selected radiographic landmarks, several numerical values, such as the sagittal location of Blumensaat’s line, the distance from Blumensaat’s line to the ACL tibial footprint, and the angle between Blumensaat’s line and the course of ACL may be influenced by the anterior tibial translation that can occur after resection of the ACL. We were concerned that, if this were to happens, the analysis of the relationship between these values and ACL tibial footprints would not be reliable. To verify for this possibility, the position of the posterior margin of the medial femoral condyle relative to the posterior margin of the medial tibial plateau was measured on full extension lateral radiographs, which were taken before and after resection of the ACL, respectively. The changes in position were compared using the Wilcoxon signed rank test, and no significant changes were found after resection of the ACL (before vs. after ACL resection: 4.73 mm vs. 4.71 mm, *p* = 0.317). Therefore, we confirmed that the anterior translation of the tibia did not occur after resection of the ACL based on the full extension lateral radiograph.

### Statistical analysis

Intraobserver and interobserver reliabilities for the location measurement of the ACL and selected radiographic landmarks on the radiographs were tested using single-measure intraclass correlation coefficients (ICCs). Two orthopedic surgeons (HJC, CBC) performed measurements twice on blinded radiographs with an interval of 3 weeks. The ICCs for intra- and interobserver reliabilities ranged from 0.902 to 0.989, which allowed us to have confidence in the reliability of the radiographic measurements produced by a single investigator (HJC).

The numerical values of the radiographic landmarks were summarized as the mean, standard deviation, and range. Correlations between the ACL tibial footprint location and the selected radiographic landmarks were determined using Pearson’s correlation coefficients (CCs). All statistical analyses were carried out using SPSS for Windows (version 15.0, SPSS Inc., Chicago, Illinois), and *P* values <0.05 were considered statistically significant.

## Results

The center of the ACL tibial footprint was 40.9 ± 2.0% in the Amis and Jakob’s line and ranged from 38.0% to 45.0%. The distance from the point where Blumensaat’s line meets the tibial articular surface to the center of the ACL tibial footprint was similar between cadavers; the mean distance was 7.6 mm (6.4–8.7 mm), with a difference of 2.3 mm between the maximum and minimum values and a variance of 0.7 mm. Furthermore, the slope of Blumensaat’s line was nearly parallel to the course of the ACL; the angle between them was 1.7 ± 1.4° (0.0–4.1°). The mean distance from the apex of the tibial eminence to the center of the ACL tibial footprint was 13.5 mm (9.4–19.1 mm). The distance varied greatly between cadavers, with a difference of 10 mm between the maximum and minimum values.

The radiographic landmark that showed a significant correlation with the ACL tibial footprint was Blumensaat’s line (Table 1). In the correlation analysis, the angle between Blumensaat’s line and the tibial articular surface was positively correlated with the ACL tibial footprint (CC: 0.704, *p* = 0.005); as the angle became steeper, the ACL tibial footprint was located more posteriorly. The angle between Blumensaat’s line and the posterior femoral cortex was negatively correlated with the ACL tibial footprint (CC: −.0.744, *p* = 0.002); as the angle decreased, the ACL tibial footprint was located more posteriorly. The sagittal location of Blumensaat’s line in the Amis and Jakob’s line was also positively correlated with the ACL tibial footprint (CC: 0.572, *p* = 0.033), whereas the location of the apex of the tibial eminence was not (*p* = 0.186).

**Table 1:** Correlation analysis between the ACL tibial footprint and other radiographic landmarks

Radiographic landmarks	Mean (range)	SD	CC^b^	*p* value^b^
Sagittal location of ACL tibial footprint^a^	40.9 (38.0 ~45.0)	2.0		
Geometry of Blumensaat’s line				
Sagittal location of Blumensaat’s line^a^	26.7 (22.0 ~34.0)	3.1	0.572	0.033
Angle between Blumensaat’s line and tibia articular margin (°)	63.9 (57.8~74.0)	4.9	0.704	0.005
Angle between Blumensaat’s line and posterior femoral cortex (°)	31.9 (26.3~37.6)	3.0	−0.744	0.002
Sagittal location of apex of tibial eminence^a^	60.3 (57.0 ~ 63.0)	1.8	0.375	0.186

^a^Data are presented as the percentage that was measured by the proportion of sagittal landmark in Amis and Jakob’s line

^b^Statistical analysis was performed using the Pearson’s correlation analysis

Abbreviation: SD (standard deviation), CC (correlation coefficient

## Discussion

This study confirmed the substantial anatomic variation between subjects with regard to the sagittal location of the ACL tibial footprints. The center of the ACL tibial footprint in our subjects was an average of 40.9% in the Amis and Jakob’s line and ranged from 38.0% to 45.0%. When comparing our findings with those of previous studies which were conducted in other populations, our ACL tibial footprint was placed in a more anterior position (Table 2) [18–27]. The reason for this slight difference between studies is not clear, but might be explained as follows. First, a difference in both sex distribution and the ethnicity of study participants may have influenced this difference. Second, the evaluation tools were quite different among the studies. Some studies used direct measurement with cryosection and MRI, while other studies utilized plain radiographs. Finally, the reference line for the description of the ACL location was different among the studies. Some studies described the ACL location as a percentage based on the anteroposterior length of the tibial articular surface, and other studies described it as a percentage based on imaginary reference lines, which defined the maximum anteroposterior length of the tibia. One study reported similar numerical values of the ACL location to the present study using the same radiographic protocol [21]. However, this does not mean that these two studies reported virtually the same location of ACL, because methods for measurement were different each other. In the previous study, measurements were taken from the tibial articular surface, whereas in the present study, measurements were based on the maximum anteroposterior length of the tibia. Therefore, there may be methodologically a few percent measurement differences between these two techniques. Additional experimental studies with unified methods would be required for more accurate assessment.

**Table 2:** Summary of previous studies of the tibial sagittal insertion of ACL

Authors	Methods	Number	Reference line (AP diameter)	ACL center^a^	AM center^a^	PL center^a^
Staubli and Rauschning [26]	cadaver	10	Staubli & Rauschning’s line^b^	41.2		
	cryosection	5	Staubli & Rauschning’s line^b^	43.3		
	MRI	23 male	Staubli & Rauschning’s line^b^	44.1		
		12 female	Staubli & Rauschning’s line^b^	43.7		
Shea et al. [25]	MRI	14 male	Staubli & Rauschning’s line^b^	43		
		7 female	Staubli & Rauschning’s line^b^	46		
Colombet et al. [18]	lateral radiographs	7	Amis & Jakob’s line		36	52
Zantop et al. [24]	lateral radiographs	20	Staubli & Rauschning’s line^b^		30	44
Doi et al. [19]	lateral radiographs	31	Amis & Jakob’s line		34.6	38.5
Pietrini et al. [23]	lateral radiographs	12	Amis & Jakob’s line		36.3	51
Iriuchishima et al. [20]	lateral radiographs	15	Staubli & Rauschning’s line^b^		31	50
Kasten et al. [27]	lateral radiographs	67	Amis & Jakob’s line		35	48
Musahl et al. [22]	lateral radiographs	8	medial tibia plateau line	46.2		
	CT	8	medial tibia plateau line	45.4		
Lintner et al. [21]	lateral radiographs	7	tibia articular surface	40		
Current study	lateral radiographs	20	Amis & Jakob’s line	40.9		

^a^Data are presented as the percentage that was measured by the proportion in the each used reference line

^b^Staubli & Rauschning’s line: The line passing through the posterior corner of the tibial plateau and perpendicular to the tibial axis

Abbreviations: AM: anteromedial, PL: posterolateral, AP: anteroposterior

There have been numerous efforts to standardize the recommended tibial tunnel placement using the radiographic reference line during ACL reconstruction. Howell and Taylor [11] showed that the ideal placement of the tibial tunnel to avoid impingement with the intercondylar notch is 44% of the anteroposterior diameter of the joint line. Using magnetic resonance arthrography, Staubli and Rauschning [26] advocated that the center of the tibial tunnel should be 44% of the mid-sagittal intercondylar line, passing through the maximum diameter of the proximal tibia. However, considering a high variability in the sagittal location of ACL tibial insertion in the reference line between populations, we believe that absolute specification in percentages from a defined reference line should not be used to determine the placement of the individual tibial tunnel.

Another important finding in this study is that the radiographic landmark that showed correlation with the location of the ACL tibial footprint in the sagittal plane is Blumensaat’s line. Correct placement of the tibial tunnel is critical for the prevention of surgically related complications, such as anterior knee pain, loss of knee extension, instability and graft impingement [9–12]. Various anatomic reference points have been used to define the position of the tibial attachment during arthroscopy. The posterior cruciate ligament has often been suggested as an intraoperative reference, with Morgan et al. [30] defining the center of the tibial footprint as lying 7 mm to 8 mm anterior to its anterior margin. Other investigators have suggested that the tibial insertion should lie on an imaginary line between the anterior horn of the lateral meniscus and the spine of the medial tubercle [31]. However, we believe that these suggested reference points may be unreliable in that they are soft tissue structures rather than fixed bony points. This study showed that the sagittal location of the ACL tibial footprint could be estimated using Blumensaat’s line. The distance between the center of the ACL tibial footprint and the point where the extension of Blumensaat’s line met the tibial articular surface was nearly constant, and the slope of Blumensaat’s line was nearly parallel to the course of the ACL. In addition, two reference angles and the sagittal location of Blumensaat’s line were significantly correlated with the location of the ACL tibial footprint. Based on these findings, estimation of sagittal locations of the ACL tibial footprint using Blumensaat’s line is potentially useful, as many authors now advocate performing anatomic ACL reconstruction.

Intraoperative fluoroscopy is an attractive option for determining the sagittal location of the ACL tibial footprints in difficult cases. In a study by Klos et al. [32] variability in graft placement was reduced significantly with the use of fluoroscopy. However, no consistent radiographic landmarks have been defined to accurately estimate the center of the ACL tibial footprints in fluoroscopy. Therefore, fluoroscopic guidelines do not accurately define the true anatomic insertion sites but only help to avoid tunnel placement outside acceptable ranges. Our findings can thus serve as a valuable reference for intra-operative tunnel positioning in fluoroscopic surgeries. However, the clinical application of our findings may have some limitations. First, this estimation using Blumensaat’s line is feasible only when there is no anterior translation of the tibia in full extension lateral radiographs. In the present study, the tibia did not subluxate anteriorly after resection of the ACL, and we confirmed this finding through the comparison of full extension radiographs taken before and after resection of the ACL. The valid explanation for this finding is that although the ACL is cut, the intact meniscus and posterior capsular structure can limit anterior tibial translation when the knee joint is fully extended. However, in clinical practice, if there is a chronic ACL injury or an injury accompanied by damage to the meniscus or the posterior capsular structure, these secondary restraints to anterior tibial translation may be impaired and anterior tibial translation may occur even though the knee is fully extended [1, 33]. In this case, the estimated ACL tibial footprint using Blumensaat’s line would be more posterior than the actual footprint. Second, the projection of Blumensaat’s line onto the tibia can be different according to the angle of knee flexion when the radiograph is taken. Any impediment to the extension of the knee joint during an operation may cause the projection of Blumensaat’s line onto the tibia to be located anteriorly in the lateral fluoroscopic image. Therefore, the estimated ACL tibial footprint would be more anterior than the actual footprint, causing a more anterior placement of the tibial tunnel. Despite these two limitations, our findings can be expected to provide valid information for assessing the appropriateness of tunnel placement after reconstruction. Furthermore, in the case of acute ACL injury without damage to other intra-articular structures, especially the meniscus and posterior capsular structure, it may be helpful to make a surgical plan for anatomic ACL reconstruction.

Because of these inherent limitations, we also analyzed other radiographic landmarks that may help to predict the sagittal location of ACL tibial footprints. The apex of the tibial eminence can be easily identified in lateral radiographs, and unlike the projection points of Blumensaat’s line, its location does not change with knee flexion. The use of this bony reference point could provide a more reliable method of accurately determining the position of the ACL tibial footprints during fluoroscopy-assisted ACL reconstruction if correlations exist between the location of the ACL tibial footprints and the apex of the tibial eminence. However, the distance from the apex of the tibial eminence to the center of the ACL tibial footprints was not consistent but varied greatly between subjects. The correlation analysis revealed that this bony landmark was not correlated with the sagittal location of the ACL tibial attachment. Therefore, determination of the location of the tibial ACL attachment by referencing the apex of the tibial eminence is likely to be unreliable.

There are some limitations of this study that must be addressed. First, anatomical variation between genders could not be considered, as the cadavers were mostly male. In addition, the cadavers were significantly older than patients normally undergoing ACL reconstruction. Even though no specimens had osteoarthritic changes, the ages of the specimens should have been considered in this anatomical study. Second, possible variations due to ethnicity could not be considered because the cadaveric knees were all from Koreans. However, the location of the ACL tibial footprint in our study was not much different from that of studies conducted in other countries. Therefore, possible differences due to ethnicity are likely to have had little effect on the analysis of our findings. Finally, our sample size was small (*n* = 20) but was similar to previous studies. To accurately estimate ACL anatomy, further studies with larger sample sizes are required.

## Conclusion

In conclusion, the center of the ACL tibial footprint was located an average of 40.9% in the Amis and Jakob’s line. However, substantial anatomical variation between subjects was found. The radiographic landmark that showed a significant correlation with the ACL tibial footprint was Blumensaat’s line. We believe that this study will contribute to more accurate tunnel placement during ACL reconstruction surgery and provide reference data for postoperative radiographic evaluation.

## Abbreviations

ACL: Anterior cruciate ligament
CCs: Correlation coefficients
ICCs: Intraclass correlation coefficients
